# The influence of the Cyclin D1 870 G>A polymorphism as an endometrial cancer risk factor

**DOI:** 10.1186/1471-2407-8-272

**Published:** 2008-09-29

**Authors:** Katie A Ashton, Anthony Proietto, Geoffrey Otton, Ian Symonds, Mark McEvoy, John Attia, Michael Gilbert, Ute Hamann, Rodney J Scott

**Affiliations:** 1 Discipline of Medical Genetics, School of Biomedical Sciences, Faculty of Health, University of Newcastle, Australia and the Hunter Medical Research Institute, NSW, 2308, Australia; 2 Hunter Centre for Gynaecological Cancer, John Hunter Hospital, Newcastle, NSW, 2305, Australia; 3 School of Medicine and Public Health, Faculty of Health, University of Newcastle, NSW, 2305, Australia; 4 Molecular Genetics of Breast Cancer, German Cancer Research Center, 69120 Heidelberg, Germany; 5 Division of Genetics, Hunter Area Pathology Service, John Hunter Hospital, Newcastle, NSW, 2305, Australia

## Abstract

**Background:**

Cyclin D1 is integral for the G1 to S phase of the cell cycle as it regulates cellular proliferation. A polymorphism in cyclin D1, 870 G>A, causes overexpression and supports uncontrollable cellular growth. This polymorphism has been associated with an increased risk of developing many cancers, including endometrial cancer.

**Methods:**

The 870 G>A polymorphisms (rs605965) in the cyclin D1 gene was genotyped in an Australian endometrial cancer case-control population including 191 cases and 291 controls using real-time PCR analysis. Genotype analysis was performed using chi-squared (χ^2^) statistics and odds ratios were calculated using unconditional logistic regression, adjusting for potential endometrial cancer risk factors.

**Results:**

Women homozygous for the variant cyclin D1 870 AA genotype showed a trend for an increased risk of developing endometrial cancer compared to those with the wild-type GG genotype, however this result was not statistically significant (OR 1.692 95% CI (0.939–3.049), p = 0.080). Moreover, the 870 G>A polymorphism was significantly associated with family history of colorectal cancer. Endometrial cancer patients with the homozygous variant AA genotype had a higher frequency of family members with colorectal cancer in comparison to endometrial cancer patients with the GG and combination of GG and GA genotypes (GG versus AA; OR 2.951, 95% CI (1.026–8.491), p = 0.045, and GG+GA versus AA; OR 2.265, 95% CI (1.048–4.894), p = 0.038, respectively).

**Conclusion:**

These results suggest that the cyclin D1 870 G>A polymorphism is possibly involved in the development of endometrial cancer. A more complex relationship was observed between this polymorphism and familial colorectal cancer.

## Background

Cyclin D1 (CCND1) is a key protein in the regulation of the cell cycle at the G1 to S phase transition, and is essential for regulation of proliferation, differentiation and transcriptional control [1]. Overexpression of cyclin D1 induces excessive cellular proliferation and is a feature of a number of cancers, including endometrial and colorectal cancer [2-6]. Specifically for endometrial cancer, numerous studies have reported increased cellular proliferation co-existing with progressive derailment of cyclin D1, leading to the progression of hyperplasia to endometrial endometriod carcinoma [7-9]. Many association studies have focused their attention to the functionally significant 870 G>A polymorphism in cyclin D1 which creates two different splice variant transcripts [10]. The normal transcript encodes exon 5 which is essential for ubiquitin-mediated proteolysis whereas the other transcript lacks the destruction box in exon 5 and increases the half life of cyclin D1 [10]. The A allele of the 870 G>A polymorphism in cyclin D1 encodes the alternate transcript and increased levels of cyclin D1 are also evident in the heterozygous state [10,11].

Previous studies have reported inconsistent findings for the cyclin D1 polymorphism and a number of different cancers. With respect to endometrial cancer, there has been one published report on the association between the cyclin D1 870 G>A polymorphism and endometrial cancer risk in Korean women [12]. Kang et al. (2005) [12] reported that endometrial cancer patients with the AA genotype had an increased risk of disease compared to carriers of the GG genotype and the combination of the GG and GA genotypes, suggestive of a recessive model for the A allele.

Endometrial cancer is the most common gynaecological malignancy in Western countries and it is important to determine the genetic variants associated with disease since the genetic basis is poorly understood. Estrogen and its metabolites have been associated with an increased risk of developing endometrial cancer due to their ability to cause DNA damaging events [13], therefore cell cycle control is integral for the recognition, repair and/or elimination of DNA damage to prevent the initiation of cancer.

The focus of this study was to examine the 870 G>A polymorphism in cyclin D1 and its association with endometrial cancer risk in Caucasians including 191 endometrial cancer cases and 291 controls.

## Methods

### Study Population

This study initially consisted of 213 consecutively recruited women with histologically confirmed endometrial cancer who presented for treatment at the Hunter Centre for Gynaecological Cancer, John Hunter Hospital, Newcastle, New South Wales, Australia between the years 1992 and 2005. Women that had additionally been diagnosed with breast cancer were excluded from this study.

The final analysis included 191 endometrial cancer patients. Data on reproductive and environmental risk factors including ethnicity, body mass index (BMI), diabetes, high blood pressure (HBP), age of diagnosis of endometrial cancer, age of menarche, age of menopause, other personal cancer history, family history of cancer (defined as cancer in the index patient plus one or more first or second degree relatives diagnosed with cancer), parity, breastfeeding, oral contraceptive use, chemotherapy, radiotherapy, hormone therapy (HT), smoking and alcohol use was collected using self reported questionnaires. Information regarding recurrence, stage, grade and histology of endometrial cancer was collected from the medical records.

The control population consisted of 291 women who were recruited between the years 2004 and 2005 for the Hunter Community Study. This study aims to identify genetic and environmental factors associated with ageing in a cohort of individuals obtained from the Hunter region, Newcastle, New South Wales, Australia. Any control that had a prior diagnosis of either breast or endometrial cancer was excluded from the study. Controls were matched to cases by sex and age.

All participants provided informed written consent prior to participation in this study. Ethics approval was obtained from the Human Research Ethics Committee, University of Newcastle and the Hunter Area Research Ethics Committee, Hunter New England Health Service, Newcastle, New South Wales, Australia.

### DNA Isolation

Genomic DNA was extracted from 10 ml EDTA blood as previously described [14].

### Molecular Analysis

Genotyping of the cyclin D1 870 G>A polymorphism (rs603965) was performed on an ABI PRISM^® ^7500 Real-Time PCR System (PE Applied Biosystems, Foster City, CA), using primers and probes from Assay-by-Demand (Applied Biosystems) (assay ID: C_744725_1). The assay was performed under universal conditions previously described [15]. The genotyping results were confirmed by a second laboratory research assistant and 5% of the samples were re-genotyped with 100% concordance. Any sample where a genotype could not be accurately assessed was re-genotyped. If it failed a second time, it was discarded from the analysis. The overall call rates were in the range from 99.7–100%.

### Statistical Analysis

Power calculations were performed using Quanto (Version 1.2.3, May 2007, . The number of cases and controls were chosen to detect a 2-fold increased risk, assuming a dominant genetic model, minor allele frequency of 6.5%, p = 0.05, 80% power and 1.52 control/case ratio. For each polymorphism, Hardy-Weinberg Equilibrium (HWE) was calculated in the control groups to check for compliance using the Institute for Human Genetics, statistics website,  (Munich, Germany). To determine differences in genotype frequencies and environmental and reproductive risk factors between the cases and controls, chi-squared (χ^2^) statistics, odds ratios (ORs) and 95% confidence intervals (CI) were calculated using unconditional logistic regression. Multivariate unconditional logistic regression was performed to determine if any risk factors altered the significance of the genotype frequency results. The risk factors taken into account were: BMI (<25 and ≥ 25 kg/m^2^) diabetes (yes/no), HBP (yes/no), HT (yes/no), personal history of cancer (yes/no), smoking (ever/never) and alcohol consumption (ever/never). Other risk factors such as age of menopause were not included in the analysis since this information was not available for the controls.

The genotype frequencies of the cyclin D1 870 G>A polymorphism was compared in the case group stratified for the following environmental and reproductive risk factors; BMI (<25 versus >= 25), age of menarche (<12 v >= 12), age of menopause (<50 versus >= 50), parity (yes/no), oral contraceptive use (ever/never), HBP (yes/no), diabetes (yes/no), personal history of ovarian, colorectal, and/or cervical cancer (yes/no), radiotherapy (yes/no), chemotherapy (yes/no), hormone therapy (yes/no), family history of uterine, breast, colorectal and/or ovarian cancer (yes/no, defined as one first or second degree relative with cancer), smoking (ever/never), alcohol (ever/never), stage of cancer, grade of cancer, histology and cancer recurrence. This analysis was performed by using chi-squared (χ^2^) analysis and ORs and 95% CIs were calculated using unconditional logistic regression.

T-tests were used to determine differences in the age of diagnosis of endometrial cancer by genotype. Kaplan Meier survival analysis was used to plot the cumulative survival versus the patient's age of diagnosis of endometrial cancer. By comparing the Kaplan-Meier survival curves for each genotype, we tested if there were differences in the age of diagnosis of endometrial cancer by genotype. The Wilcoxon's test was used to determine the significance of observations from early ages of diagnosis, log-rank test, which gives more weight to later ages and Tarone-Ware test, which is an intermediate of the two other tests were used to examine the homogeneity of the survival curves. The polymorphisms that showed a statistically significant difference between the genotypes and the age of diagnosis of endometrial cancer for all three statistical tests were further examined by a multivariate Cox regression model where a number of specific risk factors were incorporated into the analysis.

The significance levels of all tests were set at p < 0.05 and were two-sided. All statistical analysis was performed with Intercooled STATA 8.2 (Stata Corp., College Station, TX, USA), SPSS Version 15 (SPSS Inc. Chicago, IL, USA) and GraphPad Instat version 3.06 (GraphPad Software, San Diego, CA, USA).

## Results

Cases and controls were different with respect to potential endometrial cancer risk factors, including HBP, diabetes, HT, alcohol consumption, personal history of any cancer, personal history of ovarian cancer, cervical cancer and other cancers. The characteristics of the cases and controls are shown in table 1. The distributions of the cyclin D1 genotypes among the controls did not deviate from HWE.

**Table 1:** Comparison of Environmental and Reproductive Risk Factors between Cases and Controls

Risk Factor	Group	Cases n (%)	Controls n (%)	OR	95% CI	P value
High Blood Pressure (yes/no)	yes	107 (56.0)	114 (39.2)	1.978	1.366 – 2.864	p < 0.001
	no	84 (44.0)	177 (60.8)			
Diabetes (yes/no)	yes	44 (23.0)	31 (10.7)	2.51	1.519 – 4.148	p < 0.001
	no	147 (77.0)	260 (89.3)			
Hormone Therapy (yes/no)	yes	47 (24.6)	40 (13.7)	2.048	1.282 – 3.273	p = 0.003
	no	144 (75.4)	251 (86.3)			
Alcohol consumption (ever/never)	ever	92 (48.2)	228 (78.4)	0.257	0.172 – 0.382	p < 0.001
	never	99 (51.8)	63 (21.6)			
BMI	<25 kg/m^2^	34 (19.1)	72 (24.7)	0.718	0.454–1.136	p = 0.157
	>= 25 kg/m^2^	144 (80.9)	219 (75.3)			
Smoking (ever/never)	ever	52 (27.2)	68 (23.4)	1.227	0.807 – 1.865	p = 0.338
	never	139 (72.8)	223 (76.6)			
Ovarian Cancer (yes/no)	yes	7 (3.7)	1 (0.3)	11.033	1.346 – 90.403	p = 0.025
	no	184 (96.3)	290 (99.7)			
Cervical Cancer (yes/no)	yes	8 (4.2)	2 (0.7)	6.317	1.327 – 30.077	p = 0.021
	no	183 (95.8)	289 (99.3)			
History of Ovarian or Cervical Cancer (yes/no)	yes	15 (7.9)	3 (1.0)	8.182	2.335 – 28.663	p = 0.001
	no	176 (92.1)	288 (99.0)			
Personal History of Any Cancer (yes/no)	yes	51 (26.7)	28 (9.6)	3.422	2.066 – 5.667	p < 0.001
	no	140 (73.3)	263 (90.4)			
History of Skin Cancer (yes/no)	yes	20 (10.5)	19 (6.5)	1.674	0.869 – 3.228	p = 0.124
	no	171 (89.5)	272 (93.5)			
History of Bowel Cancer (yes/no)	yes	10 (5.2)	8 (2.7)	1.954	0.757 – 5.045	p = 0.166
	no	181 (94.8)	283 (97.3)			

Note: BMI not known for 13 cases.

The genotype frequencies were compared between the cases and controls for the cyclin D1 870 G>A polymorphism, however no significant differences were observed (table 2). However, there was a trend for increased risk of developing endometrial cancer for women with the AA genotype compared to those with the GG genotype (OR_adj _1.692 95% CI (0.939–3.049), p = 0.080).

**Table 2:** Association of Cyclin D1 870 G>A (rs603965) Polymorphism with Endometrial Cancer Risk

Genotype	Cases n (%)	Controls n (%)	χ^2^	OR (95% CI) and p value	
GG	49 (25.7)	93 (32.1)	p = 0.161	1.00 (reference)	
				1.262 (0.771–2.066)_adj#_	p = 0.355
				1.256 (0.815–1.938)	p = 0.302
AA	48 (25.1)	55 (19.0)		1.692 (0.939–3.049)_adj#_	p = 0.080
				1.656 (0.989–2.784)	p = 0.057
GA+AA	142 (74.3)	197 (67.9)	p = 0.131^†^	1.383 (0.869–2.201)_adj#_	p = 0.172
				1.368 (0.910–2.057)	p = 0.132
GG+GA	143 (74.9)	235 (81.0)	p = 0.107^‡^	1.458 (0.889–2.393)_adj#_	p = 0.135
				1.434 (0.924–2.226)	p = 0.108

Note: Minor Allele Frequency (MAF) 0.317.

# OR_adj_: Adjusted for BMI, HBP, diabetes, HT, personal history of cancer, smoking and alcohol use.

† p value: Wild type genotype (GG) compared to combination of heterozygous and homozygous variant genotypes (GA+AA).

‡ p value: Homozygous variant genotype (AA) compared to combination of wild type and heterozygous genotypes (GG+GA).

Kaplan-Meier survival analysis and T-tests were used to evaluate the influence of the cyclin D1 870 G>A polymorphism on the age of diagnosis of endometrial cancer. No significant differences were observed (data not shown).

Family histories of other cancers in association with the index endometrial cancer cases were identified in subsets of patients. The disease associations included first and/or second degree relatives with breast, ovarian, endometrial and colorectal cancer. No relationships between the presence of the 870 G>A SNP and endometrial cancer in association with family clusterings of breast, ovarian or endometrial cancer were observed. There was, however, a significant association between the cyclin D1 870 G>A polymorphism and family history of colorectal cancer shown in table 3. Endometrial cancer patients with the variant (AA) genotype had a higher frequency of family members with colorectal cancer compared to those with the GG and GA genotypes (GG versus AA; OR 2.951, 95% CI (1.026–8.491), p = 0.045, and GG+GA versus AA; OR 2.265, 95% CI (1.048–4.894), p = 0.038).

**Table 3:** Association of Cyclin D1 870 G>A (rs603965) Polymorphism and Family History of Colorectal Cancer in Endometrial Cancer Cases

Genotype	FH of Colorectal Cancer in Endometrial Cancer Cases (n = 191)		χ^2^	OR (95% CI) and p value	
				
	Yes n (%)	No n (%)			
GG	6 (3.1)	43 (22.5)	p = 0.084	1.00 (reference)	
GA	16 (8.4)	78 (40.8)		1.470 (0.536–4.034)	p = 0.454
AA	14 (7.3)	34 (17.8)		***2.951 (1.026–8.491)***	***p = 0.045***
GA+AA	30 (15.7)	112 (58.6)	p = 0.170^#^	1.920 (0.747–4.936)	p = 0.176
GG+GA	22 (11.5)	121 (63.4)	***p = 0.035***^†^	***2.265 (1.048–4.894)***	***p = 0.038***

Note: FH, family history.

# p value: Wild type genotype (GG) compared to combination of heterozygous and homozygous variant genotypes (GA+AA).

† p value: Homozygous variant genotype (AA) compared to combination of wild type and heterozygous genotypes (GG+GA).

In regards to the other environmental and reproductive risk factors examined in the case group, the genotype frequencies of the cyclin D1 870 G>A polymorphism showed no further significant results.

## Discussion

It is well known that endogenous and exogenous estrogen is implicated in endometrial cancer etiology since their products and metabolites have the ability to cause DNA damage. Given the importance of cell cycle control for the maintenance of genomic integrity, it is conceivable that polymorphisms in the genes that drive these processes alter the efficiency of DNA repair, leading to disease initiation. The current study took into account a series of environmental and reproductive risk factors for both the cases and controls and the results support previous epidemiological data for the listed risk factors indicating that there were no unusual environmental characteristics associated with the study population.

This study focused on the 870 G>A polymorphism in cyclin D1 and its association with endometrial cancer risk. The 870 G>A polymorphism was not significantly associated with endometrial cancer risk. However, a trend towards the AA genotype imparting an increased risk of endometrial cancer compared to the GG genotype was observed. These results support the study of Kang et al. (2005) [12] who found a significant association between the AA genotype and an increased risk in endometrial cancer development in Korean women (GG+GA versus AA, OR_adj _2.63 (1.04–6.66), p = 0.041) and suggests that this polymorphism is acting as a disease modifier. Even though we did not observe a statistically significant result, the underlying biological plausibility remains consistent between the two studies. The most likely explanation for the difference in the significance of the results between the current study and that of Kang et al. (2005) [12] is the influence of different environmental factors affecting disease risk. A number of studies have shown that overexpression of cyclin D1 is associated with endometrial cancer and that it is an early event in tumourigenesis [3,4,6-9]. One report showed that 50% (7/14) of endometrial carcinomas had cyclin D1 overexpression and that there was no immunopositive difference between these carcinomas and simple hyperplasia which is a precursor for endometrial cancer development [3].

From the study participants two main familial associations were observed, one with colorectal cancer (36 endometrial cancer patients (18.8%) with family history of colorectal cancer) and the other with breast cancer (44 endometrial cancer patients (23.0%) with family history of breast cancer). Interestingly, analysing family history of colorectal cancer in patients with endometrial cancer and the cyclin D1 870 G>A polymorphism showed that endometrial cancer patients with the AA genotype had a higher frequency of family members with colorectal cancer compared to endometrial cancer patients with the GG or GA genotype combinations.

The association between endometrial cancer and colorectal cancer is well recognised in Lynch Syndrome (or HNPCC); however, endometrial cancer associations with other malignancies are less well defined and there is little written in the literature about breast/endometrial cancer familial clustering. No association was observed between the cyclin D1 G870A SNP and endometrial/breast cancer families whereas an association was observed between this polymorphism and the increased likelihood of colorectal cancer in other family members.

There have been a number of studies that have focused on the cyclin D1 870 G>A polymorphism and colorectal cancer development however these reports are somewhat conflicting [16-18]. Some studies have reported an association between the cyclin D1 870 G>A polymorphism and the age of diagnosis of colorectal disease in HNPCC [19-21]. Endometrial cancer is the most common cancer in women with HNPCC and the second most common cancer overall in this syndrome [22], therefore the association of the cyclin D1 870 G>A polymorphism and a family history of colorectal cancer is intriguing and suggests that this polymorphism may be related to the increased risk of endometrial cancer in HNPCC.

This study has a number of strengths since it was a population-based case-control investigation that had a relatively large sample size to detect an association and detailed information of specific risk factors. However, these results must be confirmed in a larger population and more studies should be conducted on the association between the cyclin D1 870 G>A polymorphism and endometrial cancer risk. Additionally, the relationship between the cyclin D1 870 G>A polymorphism and HNPCC, specifically hMSH2 mutation carriers, should also be confirmed in further studies.

In conclusion, we have shown that the 870 G>A polymorphism in cyclin D1 may be associated with endometrial cancer risk and provided support for an association with colorectal cancer risk.

## Pre-publication history

The pre-publication history for this paper can be accessed here:


